# Mechanisms of coherent re-arrangement for long-lived spin order

**DOI:** 10.5194/mr-2-741-2021

**Published:** 2021-10-08

**Authors:** Florin Teleanu, Paul R. Vasos

**Affiliations:** 1 Extreme Light Infrastructure Nuclear Physics ELI-NP, Laser Gamma Experiments Department (LGED), Horia Hulubei National Institute for Physics and Nuclear Engineering IFIN-HH, 30 Reactorului Street, 077125 Bucharest-Măgurele, Romania; 2 Interdisciplinary School of Doctoral Studies, University of Bucharest, Blvd. Regina Elisabeta, 030018 Bucharest, Romania

## Abstract

Long-lived spin order-based approaches for magnetic resonance rely on the transition between two magnetic environments of different symmetries, one governed by the magnetic field of the spectrometer and the other where this strong magnetic field is inconsequential. Research on the excitation of magnetic-symmetry transitions in nuclear spins is a scientific field that debuted in Southampton in the year 2000. We advanced in this field carrying the baggage of pre-established directions in NMR spectroscopy. We propose to reveal herein the part of discoveries that may have been obscured by our choice to only look at them through the experience of such pre-established directions at the time. The methodological developments that are emphasised herein are the mechanisms of translation between the symmetric and non-symmetric environments with respect to the main magnetic field $B_{0}$ . More specifically, we look again thoroughly at zero-quantum rotations in the starting blocks of long-lived state populations, magnetisation transfers between hyperpolarised heteronuclei, and protons. These pulse sequences seed subsequent magnetic mechanisms that contribute to further applications. For instance, we show how some of the introduced coherence rotations were combined with classical pulse blocks to obtain two-dimensional correlations between protons and heteronuclei. We hope the pulse sequence building blocks discussed herein will open further perspectives for magnetic resonance experiments with long-lived spin order.

## 1 Introduction

This paper is an opportunity to present several magnetic resonance concepts free of particular application-specific introductions. This may allow such concepts for what they are worth simply as magnetisation transfer mechanisms and comments on their potential usefulness in further experiments. We point out that all concepts presented herein were already addressed, albeit concisely, in references (Sarkar et al., 2007; Vasos et al., 2009; Ahuja et al., 2010; Sarkar et al., 2010) or in the Supplements of these papers.

Proposing that a presentation free of application-specific introductions may reveal magnetic resonance progress to the fullest implies that the drive for traditional discipline-oriented applications may have obscured part of the concepts in the original papers. A legitimate question is whether these articles would have been accepted by the journal editors without the applications in mind or whether they would have been worth accepting. With hindsight, doubts raised by editors whether our work in Lausanne and Paris brought actual progress for applications were far more severe than any doubts regarding the soundness of the work itself. For instance, in the search for new singlet-based excitation sequences (Carravetta et al., 2004) on the route of hyperpolarised magnetisation to long-lived spin states (LLS), we were never tormented by the question “is transport of hyperpolarisation really long-lived?” (Vasos et al., 2009) (Pileio, 2020). However, “is LLS-based polarisation storage in peptides better than the mere longitudinal relaxation time constant of heteronuclei with which peptides are often isotopically enriched, $T_{1}{(}^{15}N)$ , $T_{1}{(}^{13}C)?$ ” was a harrowing question. Equally present were the doubts “are long-lived states, with their complicated excitation and sustaining mechanism, really a better way of measuring slow diffusion, slow exchange constants than heteronuclei (Ferrage et al., 2003) such as ${}^{15}N?$ ” or “are long-lived coherences (LLCs) actually a good route to improved spectral resolution in NMR?”.

When we dedicated the first of a series of papers (Sarkar et al., 2007) to Anatole Abragam along with a letter expressing our hopes that the discoveries may be useful for diffusion studies, he seized the essence of our work in his answer (mainly addressed to Geoffrey Bodenhausen): “nice to see a way of skillfully sending spins to sleep in their soft bed”, “envoyer les spins se reposer dans leur lit douillet”. The remark, thus made rhythmic by alliteration, was as concise as it was exact, since the singlet state we were searching for is magnetically inactive, i.e. the spins are “sleeping”. This commentary alone may have replaced the introduction to our original paper.

## 2 Zero-quantum rotation in the starting block of long-lived states

The structure of singlet–triplet population differences or long-lived state operators, $Q_{\mathrm{LLS}}$ ,

1 $$Q_{\mathrm{LLS}}=|S_{0}\rangle \langle S_{0}|-\frac{1}{3}(|T_{-1}\rangle \langle T_{-1}|+|T_{0}\rangle \langle T_{0}|+|T_{1}\rangle \langle T_{1}|),$$

was first discussed in formulae adapted to the zero-field magnetic structure for a two-spin system, as first created (Carravetta et al., 2004) in non-equivalent nuclei. While the preference for writing highly symmetrical long-lived states in spherical tensor operators is natural, we recurred in Lausanne, however, to Cartesian operators (Sørensen et al., 1984) in the Liouvillian space:

2 $$Q_{\mathrm{LLS}}=-N_{\mathrm{LLS}}(I_{x}S_{x}+I_{y}S_{y}+I_{z}S_{z}),$$

with $N_{\mathrm{LLS}}=4/3$ .

The form of this operator allowed us to understand the structure of coherences prone to evolution:

3 $$\text{LLS}=-\frac{4}{3}ZQ_{x}-\frac{2}{3}2I_{z}S_{z},$$

where $ZQ_{x}=\frac{1}{2}\left(2I_{x}S_{x}+2I_{y}S_{y}\right)$ is a zero-quantum coherence.

Under such a configuration, the system is immune to the scalar-coupling evolution and also to chemical shift evolution, provided the chemical shift difference between the two spins is eclipsed by ample radio-frequency radiation or by cycling the main field (Cavanagh et al., 1995).

Equation (1) proved to be one the most useful formulae in developing the general theory of long-lived states by pointing out the very nature of their extended lifetime, the population imbalance between states, or manifolds of different symmetries with respect to spin permutations (Stevanato, 2015, 2020) which cannot be interconverted by relaxation mechanisms with certain symmetries. The novelty of Eq. (3) was that it strongly connected the singlet-state explorations to research in the Ionel Solomon consecrated ( I , S ) homonuclear and heteronuclear magnetisation transfer (Solomon, 1955).

The first method of excitation for long-lived populations, developed by Levitt and collaborators (Carravetta and Levitt, 2004), worked for a pair of spins I and S provided carefully chosen delays dependent on the chemical shifts, $\nu_{I}$ and $\nu_{S}$ , were used, making a sweep through frequencies necessary to excite different pairs of coupled spins ( I , S ), ( R , K ), etc., in different experiments, just like one-dimensional magnetic resonance spectroscopy necessitated a sweep of the main field through resonance conditions for different chemical environments before the introduction of Fourier transform (Ernst and Anderson, 1966; Ernst, 2021 – Nobel Lecture). The chemical-shift dependency of long-lived states rendered impossible any two-dimensional investigations of phenomena involving two or more spin pairs or several chemical environments of spin pairs with encoded LLS, such as exchange or interaction dynamics, in the same experiment.

**Figure 1:**
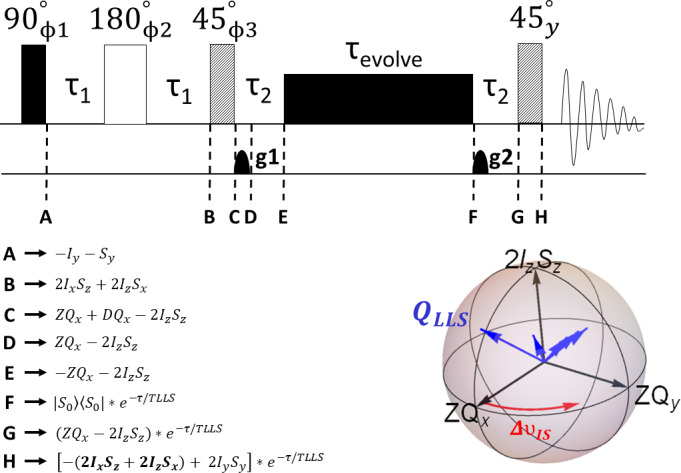
Pulse sequence adapted from a reference (Sarkar et al., 2007) showing the evolution of the density operator at different stages with an emphasis on its three projections ( $ZQ_{x}ZQ_{y}$ , $2I_{z}S_{z}$ ) between time points C and E (figure generated with SpinDynamica; Bengs and Levitt, 2018). Here, $\tau_{1}=1/(4\cdot J_{IS})$ and $\tau_{2}=1/(2\cdot \Delta \nu_{IS})$ , where $J_{IS}$ is the scalar-coupling constant (Hz) between spins I and S and $\Delta \nu_{IS}=\nu_{I}-\nu_{S}$ is the chemical shift difference (Hz) between the two spins. The phase cycling is $\phi 1=\left(x,-x\right)$ , ϕ2=x , ϕ3=2(y),2(-y) and $\phi \text{rec}=\left(x,-x,\;-x,x\right)$ .

The first concept introduced in the Lausanne paper (Sarkar et al., 2007) was the broadband excitation of singlet states. The topic may have deserved, in retrospect, a paper on its own. Our way towards broadband LLS excitation passed through zero-quantum coherences, as explained below. The first attempts to excite $Q_{\mathrm{LLS}}$ in Lausanne (Fig. 1) posed challenges regarding the evolution and relative orientation of zero-quantum coherences ( $ZQ_{x}$ , $ZQ_{y}$ ) and ZZ magnetisation ( $2I_{z}S_{z})$ .

After the first 45 ${}^{\circ }$ pulse, at point C in Fig. 1, the density operator takes the following expression:

4 $$\rho_{\mathrm{pre}\text{-}\mathrm{LLS}}^{C}=2I_{x}S_{x}-2I_{z}S_{z}=ZQ_{x}+DQ_{x}-2I_{z}S_{z}.$$

At first, we expected to induce the presence of $Q_{\mathrm{LLS}}$ at point C in this sequence, due to the presence of projections onto $Q_{\mathrm{LLS}}$ by both longitudinal two-spin order and $2I_{x}S_{x}$ . However, we realised these two contributions exactly cancel one another out, leaving us at a loss as to how to excite singlets in a broadband manner. We could have anticipated the mutual cancellation by expressing the operator at point C in the singlet–triplet basis, relevant upon application of a “sustaining” radio-frequency (rf) field:

5 $$\begin{array}{rl} \rho_{\mathrm{pre}\text{-}\mathrm{LLS}}^{C}= & -\frac{1}{2}(|T_{-1}\rangle \langle T_{-1}|+|T_{1}\rangle \langle T_{1}|-2|T_{0}\rangle \\ & \langle T_{0}|-|T_{-1}\rangle \langle T_{1}|-|T_{1}\rangle \langle T_{-1}|), \end{array}$$

indicating there was no singlet order to be found there.

By applying the pulsed field gradient $g_{1}$ , the double-quantum term dissipates and the density operator becomes

6 $$\rho_{\mathrm{pre}\text{-}\mathrm{LLS}}^{D}=(I_{x}S_{x}+I_{y}S_{y})-2I_{z}S_{z}=ZQ_{x}-2I_{z}S_{z},$$

which possesses all the components of the long-lived state (Eq. 1) but displays an opposite orientation of zero-quantum and ZZ components with equal projections onto $Q_{\mathrm{LLS}}$ . Therefore, these projections cancel each other out. To better understand this apparent conundrum, we can write the operator on the basis of singlet–triplet operators:

7 $$\begin{array}{rl} & \rho_{\mathrm{pre}\text{-}\mathrm{LLS}}^{D}=\frac{1}{2}\left(|T_{0}\rangle \langle T_{0}|-|S_{0}\rangle \langle S_{0}|\right)\\ & -\frac{1}{2}\left(|T_{-1}\rangle \langle T_{-1}|+|T_{1}\rangle \langle T_{1}|-|T_{0}\rangle \langle T_{0}|-|S_{0}\rangle \langle S_{0}|\right)\\ & =\frac{1}{2}\left(2|T_{0}\rangle \langle T_{0}|-|T_{-1}\rangle \langle T_{-1}|-|T_{1}\rangle \langle T_{1}|\right). \end{array}$$

**Figure 2:**
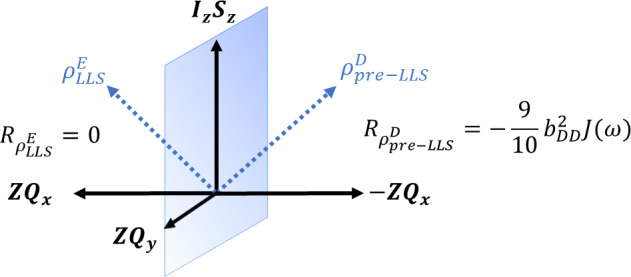
Position of coherence prior to and after zero-quantum rotation with an auto-relaxation rate constant corresponding to the density operator given by the two linear combinations of $ZQ_{x}$ and $I_{z}S_{z}$ . Only the dipolar relaxation mechanism was considered for a pair of two spins where $b_{DD}$ is the dipolar coupling constant and J(ω) is the spectral density.

The next step was figuring out how to interchange the singlet and central triplet populations in order to get the expression for LLS, a task which is not immediately obvious in this form. To do that, a reconversion in the Cartesian product basis proved fruitful: $|T_{0}\rangle \langle T_{0}|-|S_{0}\rangle \langle S_{0}|=I_{x}S_{x}+I_{y}S_{y}=ZQ_{x}$ . After several weeks of calculations, a group seminar was dedicated to the otherwise well-known evolution of $ZQ_{x}$ under a scalar-coupling interaction (Cavanagh et al., 1995). The new aspect was that the rotation axis was this time also apparent in the density operator expression, so effectively one of the constituents of spin order was rotating around the other, thus changing the relative sign of the constituent product operators $ZQ_{x}$ and $2I_{z}S_{z}$ and yielding the sought-after LLS (Fig. 2). The atmosphere in the magnetic resonance laboratory in Lausanne should be credited for a substantial contribution to the birth of these concepts. However, as a side note, the physical exercises of magnetisation succeeded in captivating more attention on paperback than in the coffee-table setting around a group-meeting white board.

The evolution of the $ZQ_{x}$ during free precession is

8 $$\begin{array}{rl} & ZQ_{x}\overset{(2\pi \nu_{I}I_{z}+2\pi \nu_{S}S_{z})\tau }{⟶}ZQ_{x}\mathrm{cos}2\pi \Delta \nu_{IS}\tau \\ & \;\;+ZQ_{y}\mathrm{sin}2\pi \Delta \nu_{IS}\tau , \end{array}$$

where $\nu_{I}$ is the Larmor frequency of spin I , $\nu_{S}$ is the Larmor frequency of spin S , $\Delta \nu_{IS}=\nu_{I}-\nu_{S}$ is the chemical shift difference (Hz) between the two spins, and $J_{IS}$ is the scalar-coupling constant (Hz) between spins I and S . Thus, after an evolution period $\tau_{2}=1/(2\Delta \upsilon )$ , the $ZQ_{x}$ will change sign such that $\frac{1}{2}(|T_{0}\rangle \langle T_{0}|-|S_{0}\rangle \langle S_{0}|)\overset{(2\pi \nu_{I}I_{z}+2\pi \nu_{S}S_{z})\tau }{⟶}\frac{1}{2}(|S_{0}\rangle \langle S_{0}|-|T_{0}\rangle \langle T_{0}|)$ . During the evolution represented in the coherence clock in Fig. 2, the right-hand side in the first quadrant is transformed into its symmetric image on the other side of the ( $2I_{z}S_{z}$ , $\;ZQ_{y}$ ) plane. The initial $\rho_{\mathrm{pre}\text{-}\mathrm{LLS}}^{D}$ and its plane-symmetric image $\rho_{\mathrm{LLS}}^{E}$ have quite different relaxation rates triggered by different symmetry rules. The density operator evolves into

9 $$\rho_{\mathrm{LLS}}^{E}=|S_{0}\rangle \langle S_{0}|-\frac{1}{2}(|T_{-1}\rangle \langle T_{-1}|+|T_{1}\rangle \langle T_{1}|).$$

A note can be made on the time dependence of the relaxation rate constant for the case of free precession of the LLS. Given the evolution of zero-quantum coherences under the chemical shift difference, the density operator, starting from $ZQ_{x}+I_{z}S_{z}$ , will have an oscillatory evolution between the two $ZQ_{x}$ and $ZQ_{y}$ as

10 $$\begin{array}{rl} ZQ_{x}+ & \;I_{z}S_{z}\overset{(2\pi \nu_{I}I_{z}+2\pi \nu_{S}S_{z})\tau }{⟶}ZQ_{x}\mathrm{cos}2\pi \Delta \nu_{IS}\tau \\ & +ZQ_{y}\mathrm{sin}2\pi \Delta \nu_{IS}\tau +I_{z}S_{z}. \end{array}$$

Considering a relaxation superoperator only for the dipolar interaction between two coupled spins,

11 $$\hat{\Gamma }=-\frac{6}{5}b_{DD}^{2}\sum_{m=-2}^{2}{\left(-1\right)}^{m}J\left(m\times \omega \right)\left[T_{2,m}\left[T_{2,-m}\_\right]\right],$$

where $b_{DD}$ is the dipolar coupling constant, J(ω) is the spectral density, and $T_{2,m}$ are the spherical tensor spin operators of rank 2, the computed relaxation rate constant for the density operator $ZQ_{x}\mathrm{cos}2\pi \Delta \nu_{IS}\tau +ZQ_{y}\mathrm{sin}2\pi \Delta \nu_{IS}\tau +I_{z}S_{z}$ is

12 $$\begin{array}{rl} R(\tau )= & -\frac{6}{5}b_{DD}^{2}(\left(1+\mathrm{cos}2\pi \Delta \nu_{IS}\tau \right)\cdot J(0)\\ & +3\cdot J(\omega ))\cdot (\mathrm{sin}\frac{2\pi \Delta \nu_{IS}\tau }{2}{)}^{2}. \end{array}$$

Thus, only for $\tau =\frac{2n}{2\pi \Delta \nu_{IS}}$ in the starting blocks is the relaxation rate constant of the obtained state optimally low.

Juggling with operators in order to drive the spin system in its “soft bed”, we realised we should always look at nature from various perspectives. We learned that if only one of the longitudinal two-spin-order and zero-quantum components could be selected at time point C (Fig. 1), $Q_{\mathrm{LLS}}$ would have been present already. For instance, ZZ magnetisation alone projects onto the long-lived state, given that $2I_{z}S_{z}=\frac{1}{2}(|T_{-1}\rangle \langle T_{-1}|+|T_{1}\rangle \langle T_{1}|-|T_{0}\rangle \langle T_{0}|-|S_{0}\rangle \langle S_{0}|)$ . Thus, during the sustaining period, only the singlet population will survive for a period much longer than longitudinal magnetisation. In order to do so, we employed a Thrippleton–Keeler (Thrippleton and Keeler, 2003) filter to wipe out the troubling zero- and double-quantum coherences and obtained a singlet state with an amplitude 2 times lower than using both zero-quantum and ZZ magnetisation. Other groups employed the so-called “pseudo singlet order” (Pileio, 2017), which is just $ZQ_{x}=\frac{1}{2}\left(|T_{0}\rangle \langle T_{0}|-|S_{0}\rangle \langle S_{0}|\right)$ , as the source of a long-lived state, obtaining similar results.

Though broadband excitation of singlet states would have deserved publication as a discovery in its own right, we were cautious to avoid publication of our research in slices of “salami science” (Sweedler, 2019). However, this advance proved relevant for the advancement of long-lived state order (Pileio, 2020; Teleanu et al., 2021; Pileio, 2017; Bengs et al., 2020) and was more challenging to obtain than the two-dimensional spectroscopy application for the study of singlet-state-based exchange we describe in the same paper (SS-EXSY) (Sarkar et al., 2007). In the tradition of finding low-key names for sequences such as “INEPT” (Morris and Freeman, 1979) or “INADEQUATE” (Bax et al., 1980), we could have named the zero-quantum rotation block of the pulse sequence in Fig. 1 a “( ZZ -)ZEROTATION”.

As singlet-based applications aim to store magnetisation for ever-longer time periods, the most adapted systems to this purpose, quasi-equivalent spin pairs with J couplings far overweighing the differences between the chemical shifts of the components, became increasingly studied. Spin dynamics that shift the magnetisation of the two spins differentially to create the singlet state are particularly challenging in such systems.

**Figure 3:**
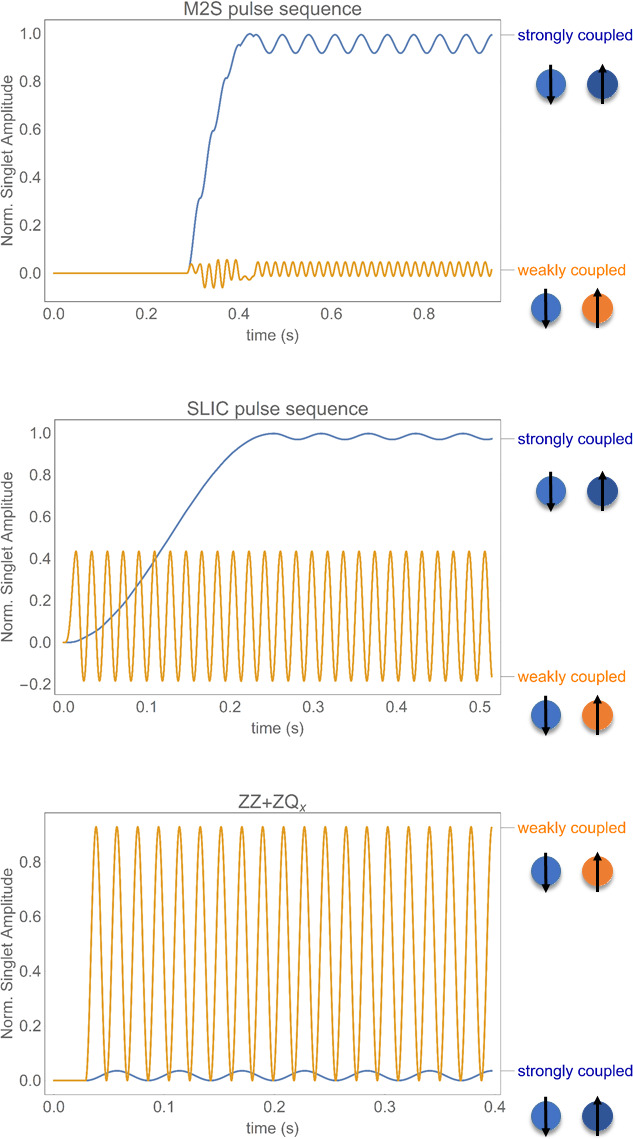
Numerical simulations using SpinDynamica outlining the efficiency of singlet polarisation for M2S, SLIC and $ZZ+ZQ_{x}$ pulse sequences in a two-spin system (by projecting the density operator during the pulse sequence onto the singlet population). The weakly coupled system is described by $\left\{\Delta \nu_{IS}=50\;\mathrm{Hz};J_{IS}=17.4\;\mathrm{Hz}\right\}$ , while the strongly coupled system features $\left\{\Delta \nu_{IS}=2.8\;\mathrm{Hz};J_{IS}=17.4\;\mathrm{Hz}\right\}$ . For each scenario, only the coherent evolution was considered in simulations (no relaxation dampening of the amplitude of source operators or LLS is taken into account). The excitation period is followed by a free-precession evolution after the maximum amplitude for the singlet population was reached.

Pulse sequences of particular interest include the magnetisation to singlet (Tayler and Levitt, 2011) and spin-lock-induced crossover (DeVience et al., 2013). These methods are suited for strongly coupled spins where the scalar coupling is larger than the chemical shift difference, while the pulse sequence described in Fig. 2, which we identify herein as “ $ZZ+ZQ_{x}$ ” (Sarkar et al., 2007), performs better in terms of long-lived state excitation in the weakly coupled regime. Several attempts to efficiently excite singlet order on broader domains of coupling regimes have been recently devised (Bengs et al., 2020; Mamone et al., 2020). Figure 3 depicts numerical simulations performed with SpinDynamica (Bengs and Levitt, 2018) for singlet population excitation using the aforementioned pulse sequences for both weakly and strongly coupled regimes, outlining the difference in excitation efficiency.

## 3 Heteronuclei or proton long-lived states for conserving hyperpolarisation

In order to maximise the magnetisation lifetime, heteronuclear longitudinal spin order (mainly on carbon-13) can be excited and used during evolution periods in both room-temperature (Bermel et al., 2005b; Richter et al., 2010) and hyperpolarised NMR. We strove to also preserve hyperpolarisation on a pair of hydrogens entwined in a long-lived state (Vasos et al., 2009). Since the invention of dissolution–dynamic nuclear polarisation (dissolution–DNP) (Ardenkjær-Larsen et al., 2003; Comment et al., 2008; Balzan et al., 2016) and its development in Lausanne by the team of Arnaud Comment, Sami Jannin and Jac van der Klink in the Functional Imaging Laboratory at EPFL (Ardenkjær-Larsen et al., 2003; Comment et al., 2008; Balzan et al., 2016), the topic has been associated with our research due to its conjunction with long-lived spin order (Ardenkjær-Larsen et al., 2003; Comment et al., 2008; Balzan et al., 2016).

The preservation of hyperpolarised magnetisation obtained by dissolution–DNP in long-lived states raised fewer challenges than the comparison of the LLS with heteronuclear lifetimes in terms of performance as polarisation batteries. The hyperpolarised magnetisation in samples stemming from a polariser such as the one developed in Lausanne and the similar one installed as the first dissolution–DNP system in France (Ardenkjær-Larsen et al., 2003; Comment et al., 2008; Balzan et al., 2016) followed intently the rf pulses in our high-resolution magnets. Inhomogeneities due to fast dissolution and injection could be tamed to run pulse sequences for LLS excitation and decoding and to observe the signal. To us, the main issue remained that the benefits of a procedure consisting of $Q_{\mathrm{LLS}}$ excitation on protons compared to simply preserving hyperpolarised magnetisation in heteronuclei had to be carefully considered. We scrutinised this issue in terms of magnetisation lifetimes in the given conditions (room temperature, molecular size) as well as in the case of extreme molecular sizes or crowding of the environment and came to the conclusion that proton-based LLS were valuable for storing magnetisation even when the molecules contained isotope-enriched heteronuclei like ${}^{15}N$ or ${}^{13}C$ . In this analysis, we were inspired by similar comparisons between proton and heteronuclear magnetisation carried out for relaxation rates of heteronuclei and protons in large or paramagnetic proteins (Bermel et al., 2005a).

Journal editors understood the interest of the research topic, and the papers we sent for publication encountered no uphill Sisyphean battles (Molinié and Bodenhausen, 2013; The Myth of Sisyphus – Wikiwand, https://www.wikiwand.com/en/Sisyphus, last access: 21 September 2021, Evslin, 2006). The only significant delays in publication we incurred were for a paper submitted directly to a specialised journal that discussed results obtained using our freshly installed DNP system at the time (Balzan et al., 2017).

**Figure 4:**
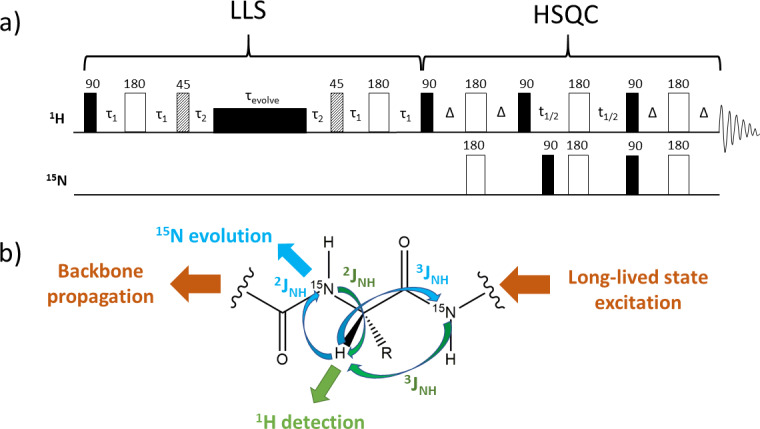
**(a)** Pulse sequence designed to generate a two-dimensional correlation spectrum between ${}^{1}H$ and ${}^{15}N$ via scalar coupling of the type ${}^{2}J_{\mathrm{NH}}$ and ${}^{3}J_{\mathrm{NH}}$ starting from a proton long-lived state. **(b)** Schematic representation of polarisation transfer along a protein's backbone which generates a two-dimensional correlation spectrum via the ${}^{2}J_{\mathrm{NH}}$ and ${}^{3}J_{\mathrm{NH}}$ coupling constants. Protons are excited via the first part of the pulse sequence from **(a)** into a long-lived state spin order. Figure adapted from the reference Fernandes et al. (2013a).

Potential applications of symmetry-adapted states as magnetisation reservoirs for various two-dimensional experiments led us to investigate the polarisation transfer from long-lived states of protons towards heteronuclei across proteins' backbones (Fig. 4) (Fernandes et al., 2013a, b). The particular relaxation rates of LLS reflect conformational exchange and act as probes for unravelling proteins' inner dynamics, while two-dimensional correlations (e.g. ${}^{1}H{-}^{15}N$ ) help disentangle complex spectra, which is acutely needed in the analysis of unfolded or intrinsically disordered proteins. The field of two-dimensional heteronuclear experiments using long-lived spin order (Fig. 4) is likely to develop further. As the field advances, long-lived state-based explorations of inter-molecular (Stavarache et al., 2017; Bornet et al., 2011) and intra-molecular (Ahuja et al., 2007; Tayler et al., 2010) interactions become important, and so becomes spectral resolution for the study of systems of increasing complexity.

## 4 Long-lived coherences, eppur' si muove

Aware of the potential of long magnetisation lifetimes for line narrowing in NMR, we strove to obtain some type of magnetisation akin to LLS to rotate. Long lifetimes of magnetisation had been traded for spectral resolution, e.g. for ${}^{1}H{-}^{15}N$ pairs in cross-correlated relaxation experiments and for ${}^{15}N$ heteronuclei for narrowing spectroscopic lines (Goldman, 1984; Pervushin et al., 1997; Vasos et al., 2006).

Our search for “moving” long-lived configurations first involved complicated coherences in alanine, serine, and other molecules with up to five coupled spins (Ahuja et al., 2009). When we finally resorted to our favourite paired Gly aliphatic protons of AlaGly, the $I_{x}$ – $S_{x}$ configuration was deduced from the diagonalised Liouvillian (Carravetta and Levitt, 2005). The next hurdle appeared in fitting the exotic long-lived states with products of oscillating and decaying functions and translating them to signals in two-dimensional spectra via Fourier transform-adapted spectroscopy (prior experience of non-conventional heteronuclear two-dimensional experiments (Bertini et al., 2004; Vasos et al., 2005) helped at this point). The simple scheme involving a 180 ${}^{\circ }$ pulse for creating a $Q_{\mathrm{LLC}}$ observable in the indirect dimension of a two-dimensional experiment, where

13 $$\begin{array}{rl} Q_{\mathrm{LLC}}= & \;(I_{x}-S_{x})\mathrm{cos}(2\pi J_{IS}\tau )+(2I_{y}S_{z}-2I_{z}S_{y})\\ & \mathrm{sin}(2\pi J_{IS}\tau ), \end{array}$$

was further refined by different approaches (Fig. S1 in the Supplement), of which the most advanced generates coherences with spins pointing in opposite ways in molecules with almost-equivalent nuclei (Sheberstov et al., 2019).

When we sought long-lived coherences in high fields, we were not aware yet that the contemporary work on extremely low-frequency oscillations (Pileio et al., 2009) in low magnetic fields developed at Southampton involved, practically, the same operators.

Just as in the case of LLS for hyperpolarisation safekeeping was compared to heteronuclear storage, once LLCs were developed, comparisons with zero-quantum coherences came to mind. For the case of two J -coupled, non-equivalent spins, I and S , the evolution of $\rho_{1}(0)=I_{x}-S_{x}$ and $\rho_{ZQx}(0)=I_{x}S_{x}+I_{y}S_{y}$ during free precession (without any radio-frequency “sustaining” applied) is given by

14 $$\begin{array}{rl} \rho_{1}(\tau )=[ & \left(I_{x}-S_{x})\mathrm{cos}(\pi J_{IS}\tau )+(2I_{y}S_{z}-2I_{z}S_{y}\right)\\ & \mathrm{sin}(\pi J_{IS}\tau )]\mathrm{cos}(\frac{2\pi \Delta \nu_{IS}\tau }{2})\\ & -[(I_{y}+S_{y})\mathrm{cos}(\pi J_{IS}\tau )\\ & -(2I_{x}S_{z}+2I_{z}S_{x})\mathrm{sin}(\pi J_{IS}\tau )]\\ & \mathrm{sin}(\frac{2\pi \Delta \nu_{IS}\tau }{2}), \end{array}$$

15 $$\begin{array}{rl} \rho_{ZQx}\left(\tau \right)= & \left(I_{x}S_{x}+I_{y}S_{y}\right)\mathrm{cos}\left(2\pi \Delta \nu_{IS}\tau \right)\\ & +\left(I_{y}S_{x}-I_{x}S_{y}\right)\mathrm{sin}\left(2\pi \Delta \nu_{IS}\tau \right)\\ & =ZQx\mathrm{cos}(2\pi \Delta \nu_{IS}\tau )\\ & +ZQy\mathrm{sin}(2\pi \Delta \nu_{IS}\tau ), \end{array}$$

while in the presence of rf fields with the carrier placed in the middle of their offsets ( $\upsilon_{1}=-\Delta \upsilon_{IS}/2$ and $\upsilon_{2}=\Delta \upsilon_{IS}/2)$ and an amplitude $\upsilon_{1}\gg \Delta \upsilon_{IS}$ , the evolutions for the LLC and $ZQ_{x}$ are

16 $$\begin{array}{rl} \rho_{LLC}(\tau )= & \;(I_{x}-S_{x})\mathrm{cos}(2\pi J_{IS}\tau )+(I_{y}S_{z}-I_{z}S_{y})\\ & \mathrm{sin}(2\pi J_{IS}\tau ), \end{array}$$

17 $$\begin{array}{rl} \rho_{ZQx}(\tau )= & I_{x}S_{x}+I_{y}S_{y}*\mathrm{cos}{\left(2\pi \upsilon_{1}\tau \right)}^{2}\\ & +I_{z}S_{z}*\mathrm{sin}{\left(2\pi \upsilon_{1}\tau \right)}^{2}+(I_{y}S_{z}+I_{z}S_{y})\\ & \mathrm{sin}\left(2\pi \upsilon_{1}\tau \right)\mathrm{cos}\left(2\pi \upsilon_{1}\tau \right). \end{array}$$

**Figure 5:**
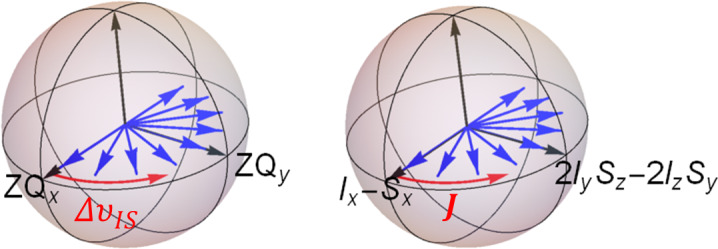
Evolution of zero-quantum coherences during free precession with an oscillating frequency of $\Delta \upsilon_{IS}$ and evolution of long-lived coherences during a sustaining period with an oscillating frequency equal to the scalar-coupling constant $J_{IS}$ .

In the absence of sustaining rf fields, differences of single-quantum transverse coherences, the source of LLCs, evolve under the chemical shift difference and the scalar coupling, while ZQ s are immune to the latter (Cavanagh et al., 1995). In the second scenario, LLCs evolve only under the effect of J coupling (Sarkar et al., 2010), oscillating between in-phase $\left(I_{x}-S_{x}\right)$ and anti-phase $\left(2I_{y}S_{z}-2I_{z}S_{y}\right)$ , with the coherence order equal to 1. Thus, the scalar-coupling evolution sets LLCs apart from ZQs (Fig. 5).

Broadband excitation of LLCs in molecules with broadly different J couplings and chemical shifts is still a challenge, despite the progress. We explored part of the territory by exciting with a series of selective 180 ${}^{\circ }$ pulses (Sarkar et al., 2011) and by sustaining with various pulse trains (Sadet et al., 2014), but we can safely say that LLCs benefit from their simple and parametric-free excitation scheme which consists of a selective π pulse and non-selective π/2 hard pulse followed by spin lock.

## 5 Conclusions

We present from today's perspective several challenging aspects in the introduction of coherent dynamics designed to render spin order resilient to Chronos' decrees. The focus is placed on zero-quantum inversion with respect to longitudinal two-spin order in homonuclear spin systems. Numerical simulations outlining the efficiency of different pulse sequences in creating long-lived states in different coupling regimes are discussed. The presented methods are delineated in a manner designed to render them useful as building blocks in further applications.

## Supplement

10.5194/mr-2-741-2021-supplement The supplement related to this article is available online at: https://doi.org/10.5194/mr-2-741-2021-supplement.

## Data Availability

The only data are the notebooks used for simulation which were deposited on ResearchGate as https://doi.org/10.13140/RG.2.2.12614.40008 (Teleanu, 2021). The link was also added in the Supplement.

## Code availability

The Mathematica notebook used for the simulations is provided as a Supplement.
