# Supplementary material for: Mechanisms of coherent re-arrangement for long-lived spin order
Source: Magn Reson (Gott). 2021 Oct 8;2(2):741–9. doi: 10.5194/mr-2-741-2021 (PMC10539845; doi:10.5194/mr-2-741-2021)
Supplement: The supplement related to this article is available online at: https://doi.org/10.5194/mr-2-741-2021-supplement. [file mr-2-741-supplement.zip › mr-2-741-2021-supplement-title-page.pdf]

Supplement of Magn. Reson., 2, 741–749, 2021
https://doi.org/10.5194/mr-2-741-2021-supplement
© Author(s) 2021. CC BY 4.0 License.

Supplement of

Mechanisms of coherent re-arrangement for long-lived spin order

Florin Teleanu and Paul R. Vasos

Correspondence to: Paul R. Vasos (paul.vasos@eli-np.ro)

- mr-2-741-2021-supplement-title-page.pdf
- M2S_SLIC_ZZZQx_comparison_weak_strong_coupling.nb
- Supplement.pdf

The copyright of individual parts of the supplement might differ from the article licence.
