# Supplementary material for: Mechanisms of coherent re-arrangement for long-lived spin order
Source: Magn Reson (Gott). 2021 Oct 8;2(2):741–9. doi: 10.5194/mr-2-741-2021 (PMC10539845; doi:10.5194/mr-2-741-2021)
Supplement: The supplement related to this article is available online at: https://doi.org/10.5194/mr-2-741-2021-supplement. [file mr-2-741-supplement.zip › Supplement.pdf]

Table of Contents:

1. **Figure S1:** Experimental methods known so far for converting thermal equilibrium nuclear spin polarization into long-lived coherences
2. **Mathematica Notebooks:** link to the numerical simulations using SpinDynamica to compare the singlet polarization efficiency of M2S, SLIC and ZZ+ZQ_x in different coupling regimes.

1. **Figure S1:** Experimental methods known so far for converting thermal equilibrium nuclear spin polarization into long-lived coherences

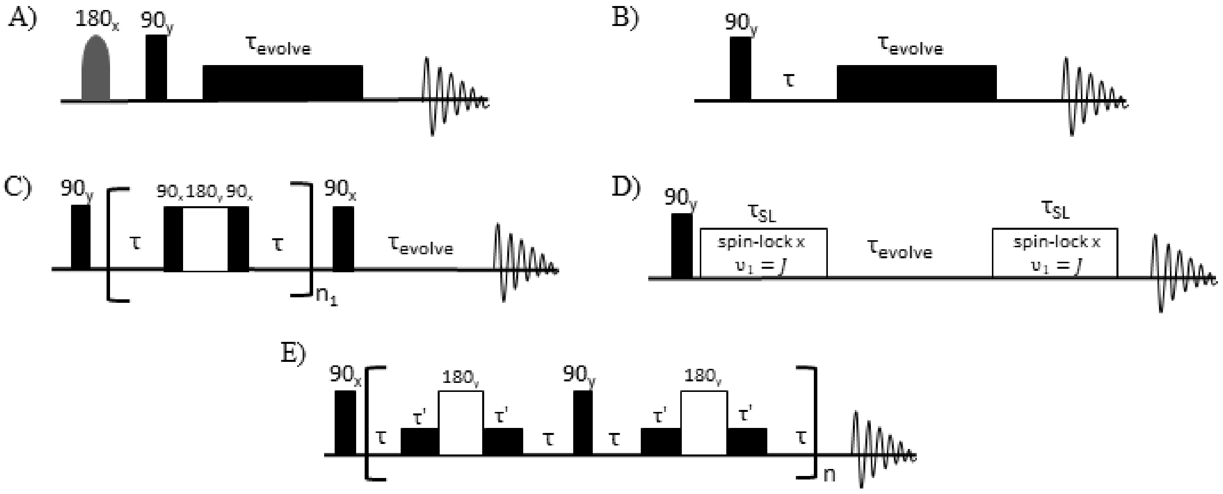

Figure S1. Experimental methods known so far for converting thermal equilibrium nuclear spin polarization into long-lived coherences. A) The first pulse sequence used to excite LLC, with a selective π-pulse followed by a non-selective π/2-pulse and spin-lock(Sarkar et al., 2010); B) a similar pulse sequence, but the relative orientation of spin angular momentum operators is given by an evolution period under the effect of different chemical shifts ($\tau = 1/2\Delta\upsilon$) ; C) First half of the M2S pulse sequences, with trains of pulses whose repetition number n_1 is optimized as a function of scalar coupling and chemical shift difference(Tayler & Levitt, 2011b); D) SLIC pulse sequence that also excite LLC during the spin-lock with amplitude equal to the scalar coupling between the two nuclei[5] (sequences C and D are particularly adapted for molecules with 'quasi-equivalent' spins, i.e., where differences in chemical shifts between the spins involved are less than the J-couplings between these spins); E) Improved LLC pulse sequence with a refocussing π/2-pulse train that allows for a better determination of the coherence's lifetime ($\tau = 1/2\Delta\upsilon$; $\tau' = 1/4J$) (Singh & Kurur, 2015).

2. **Mathematica Notebooks:** link to the numerical simulations using SpinDynamica to compare the singlet polarization efficiency of M2S, SLIC and $ZZ+ZQ_x$ in different coupling regimes.

   The Mathematica notebook can be accessed free of charge at the following link:

   DOI: 10.13140/RG.2.2.12614.40008
